# Enhancing the Antiviral Potency of Nucleobases for Potential Broad-Spectrum Antiviral Therapies

**DOI:** 10.3390/v13122508

**Published:** 2021-12-14

**Authors:** Ruben Soto-Acosta, Tiffany C. Edwards, Christine D. Dreis, Venkatramana D. Krishna, Maxim C-J. Cheeran, Li Qiu, Jiashu Xie, Laurent F. Bonnac, Robert J. Geraghty

**Affiliations:** 1 Center for Drug Design, College of Pharmacy, University of Minnesota, Minneapolis, MN 55455, USA; rsotoaco@umn.edu (R.S.-A.); edwa0373@umn.edu (T.C.E.); dreis020@umn.edu (C.D.D.); liqiu2017@gmail.com (L.Q.); jxie@umn.edu (J.X.); 2 Department of Veterinary Population Medicine, College of Veterinary Medicine, University of Minnesota, St. Paul, MN 55108, USA; vdivanak@umn.edu (V.D.K.); cheeran@umn.edu (M.C.-J.C.)

**Keywords:** nucleobase, antiviral, favipiravir, broad-spectrum, emerging viruses, antimetabolite, synergy

## Abstract

Broad-spectrum antiviral therapies hold promise as a first-line defense against emerging viruses by blunting illness severity and spread until vaccines and virus-specific antivirals are developed. The nucleobase favipiravir, often discussed as a broad-spectrum inhibitor, was not effective in recent clinical trials involving patients infected with Ebola virus or SARS-CoV-2. A drawback of favipiravir use is its rapid clearance before conversion to its active nucleoside-5′-triphosphate form. In this work, we report a synergistic reduction of flavivirus (dengue, Zika), orthomyxovirus (influenza A), and coronavirus (HCoV-OC43 and SARS-CoV-2) replication when the nucleobases favipiravir or T-1105 were combined with the antimetabolite 6-methylmercaptopurine riboside (6MMPr). The 6MMPr/T-1105 combination increased the C-U and G-A mutation frequency compared to treatment with T-1105 or 6MMPr alone. A further analysis revealed that the 6MMPr/T-1105 co-treatment reduced cellular purine nucleotide triphosphate synthesis and increased conversion of the antiviral nucleobase to its nucleoside-5′-monophosphate, -diphosphate, and -triphosphate forms. The 6MMPr co-treatment specifically increased production of the active antiviral form of the nucleobases (but not corresponding nucleosides) while also reducing levels of competing cellular NTPs to produce the synergistic effect. This in-depth work establishes a foundation for development of small molecules as possible co-treatments with nucleobases like favipiravir in response to emerging RNA virus infections.

## 1. Introduction

Broad-spectrum antiviral therapies hold promise as a critical defense against emerging viruses by controlling the severity and spread of infections until vaccines and virus-specific antivirals are developed. One antiviral compound often discussed as a broad-spectrum inhibitor is the nucleobase favipiravir [1]. A nucleobase is the base of a nucleoside without the ribose and phosphate moieties. Favipiravir displays broad-spectrum antiviral activity against RNA viruses in cell culture with a good safety profile [2,3] and has been evaluated as a possible therapeutic for influenza A virus (IAV) [2], severe acute respiratory syndrome coronavirus 2 (SARS-CoV-2) [4], and Ebola virus [5]. Favipiravir has been approved for government stockpiling and as a therapeutic tool against possibly severe influenza caused by emerging or re-emerging virus strains in Japan and Taiwan [2]. However, recent Ebola virus and SARS-CoV-2 clinical trials showed no clear antiviral potency or health benefits [6,7,8,9].

Evidence suggests that favipiravir, and its defluorinated analog T-1105, exert antiviral effects through lethal mutagenesis [10,11,12,13], and possibly chain termination [14,15]. The mutagenesis occurs via ambiguous base-pairing during viral RNA replication after insertion of the active nucleoside triphosphate form of favipiravir or T-1105 into viral RNA. RNA virus genome replication is error-prone due to high processivity rates and lack of effective proofreading [16,17]. The elevated error-rate allows the virus to adapt to its surroundings including developing mutations for resistance to antiviral therapies. However, even a slight increase in the error-rate can greatly reduce infectious virus production due to the increased frequency of deleterious mutations [18,19]. The concept of enhanced, or lethal, mutagenesis has been proposed as an important therapeutic antiviral approach [20].

In order to be incorporated into the viral RNA by the viral RNA-dependent RNA polymerase (RdRP) and exert an antiviral effect, nucleobases such as favipiravir require the addition of ribose and triphosphate moieties. An initial step is the bio-conversion of the nucleobase to the corresponding ribonucleoside-5′-monophosphate (RMP) in a single step by enzyme-mediated condensation of the 5-phosphoribosyl-1-pyrophosphate (PRPP) with the nucleobase [21,22]. Cellular kinases catalyze further phosphorylation to produce the active triphosphate form. The efficiency of favipiravir or T-1105 conversion to the RMP form is cell line-dependent and the subsequent conversion from RMP to diphosphate (RDP) and triphosphate (RTP) forms is not efficient [23]. Another drawback for favipiravir is its rapid clearance before conversion to its active form [9,24]. Strategies to enhance the efficiency of nucleobase conversion to nucleoside triphosphate (NTP) may result in more potent and effective nucleobase antivirals in vivo and reduce the amounts of drugs needed for effective treatment. In addition, a temporary reduction in normal cellular NTP pools, possibly by the co-administration of a cellular NTP synthesis inhibitor (antimetabolite), may enhance the selection of the antiviral nucleotide by the viral RdRP and increase antiviral effects.

Nucleobase/antimetabolite combinations have been evaluated against viruses in cell culture and animal models, including multiple studies using ribavirin as an antimetabolite with differing nucleobases/nucleosides [25,26,27]. However, in-depth studies to demonstrate the mechanism of antiviral synergism for published combinations are clearly lacking. Without a detailed understanding of combination effects, improving the antiviral responses of known combinations and identifying novel combinations remain challenging.

In this work, we report a synergistic reduction of flavivirus (dengue and Zika viruses), orthomyxovirus (IAV), and coronavirus (human coronavirus OC43 and SARS-CoV-2) replication when the nucleobases favipiravir or T-1105 were combined with the antimetabolite 6-methylmercaptopurine riboside (6MMPr). The 6MMPr/T-1105 combination increased the C-U and G-A mutation frequency compared to treatment with T-1105 alone, contributing to the enhanced antiviral activity. Further analysis revealed that the 6MMPr/T-1105 co-treatment resulted in an accumulation of critical bioconversion co-factor PRPP, a reduction in cellular purine NTP synthesis, and an increased conversion of the antiviral nucleobase to its RMP, RDP, and RTP forms compared to T-1105 alone. The 6MMPr synergistic effects were specific for nucleobases and not observed when combined with the nucleoside version of T-1105, T-1106. Therefore, the 6MMPr co-treatment specifically increased production of the active antiviral form of the studied nucleobases while also reducing levels of competing cellular NTPs to produce the synergistic effect. This work provides a novel, in-depth study of the mechanisms behind the synergistic enhancement of nucleobase antiviral activity by antimetabolites. Nucleobase/antimetabolite combinations warrant further exploration as potential therapies for emerging viral outbreaks.

## 2. Materials and Methods

### 2.1. Cell Lines, Viruses, and Compounds

Huh7 cells were obtained from JCRB Cell Bank (Japan). Vero and Vero-E6 cells were obtained from ATCC, Manassas, VA, USA, (CRL-81 and CRL-1586, respectively). Vero cells were maintained in Dulbecco’s modified Eagle’s medium (DMEM) supplemented with 10% fetal bovine serum (FBS), 100 IU streptomycin/penicillin per mL, and 10 μg/mL plasmocin (InvivoGen, San Diego, CA, USA). Huh7 cells were maintained in minimum essential medium (MEM) supplemented with 10% FBS, 100 IU streptomycin/penicillin per mL, 1 mM sodium pyruvate, 1X non-essential amino acids (NEA), 5 μg/mL plasmocin (InvivoGen), and 1X Glutamax. Vero-E6 cells were maintained in DMEM supplemented with 5% FBS, 100 IU streptomycin/penicillin per mL, 1 mM sodium pyruvate, 1X NEA, 5 μg/mL plasmocin (InvivoGen), and 1X Glutamax. Human foreskin fibroblasts (HFFs) were maintained in Dulbecco’s modified Eagle’s medium (DMEM) supplemented with 10% fetal bovine serum (FBS), 100 IU streptomycin/penicillin per mL, and 10 μg/mL plasmocin (InvivoGen).

Dengue virus (DENV) type 2 New Guinea C strain (ATCC VR-1584) was generated in C6/36 mosquito cell cultures (ATCC CRL-1660) as described [13]. SARS-CoV-2 isolate USA-WA1/2020 (NR-52281) was obtained through BEI Resources and propagated in Vero-E6 cells. ZIKV H/PAN/2015/CDC-259359 (BEI Resources NR-50219) stocks were generated in C6/36 mosquito cell cultures (ATCC CRL-1660) as described [13]. IAV A/WS/33 (H1N1) was obtained from ATCC (VR-825) and stocks were prepared as described [28]. HCoV-OC43 was a kind gift from J. Wang (University of Arizona) and stocks were prepared in Vero cells. Vero cells in 175 mL flasks (85% confluence) were inoculated with HCoV-OC43 at MOI of 0.01 in 10 mL of infection medium (MEM with 1% FBS, 100 IU streptomycin/penicillin per mL, and 10 mM HEPES). After 2 h, an additional 10 mL of infection medium was added. The supernatant was collected after 7 days (50% CPE) and precleared by centrifugation at 3000 RPM for 15 min (4 °C). Titer (PFU/mL) was estimated by plaque assay.

Cell lines and viral reagents used for experiments in the Supplementary Materials are described in the Supplementary Materials and Methods.

Compounds T-1105, favipiravir (T-705), 6MMPr, and T-1106 were purchased from Alfa Aesar (Haverhill, MA, USA), Asta Tech (Bristol, PA, USA), Millipore Sigma (Burlington, MA, USA), and Biosynth Carbosynth (Newbury, UK), respectively. All compounds were resuspended in DMSO to a 20 mM stock solution prior to diluting in DMSO and then into a cell culture medium for a final DMSO concentration of 0.5% in all experiments. All compounds and dilutions were stored at −20 °C.

### 2.2. Cell Viability Assay

Cell viability determination for cell lines was performed following the same experimental design conditions (cells, cell number, compound concentrations, and timing) of its respective antiviral assay. Viability assays were performed using the 3-(4,5-dimethylthiazol-2-yl)-5-(3-carboxymethoxyphenyl)-2-(4-sulfophenyl)-2H-tetrazolium (MTS)-based tetrazolium reduction CellTiter 96 Aqueous Non-Radioactive cell proliferation assay (Promega G5430, Madison, WI, USA) as described [13]. The data obtained were used to calculate the CC_50_ value using GraphPad Prism 8 software (GraphPad Software, LLC, San Diego, CA, USA).

### 2.3. Virus-Based Assays and Viral Titer Analysis

Huh7 cells (1.5 × 10^5^ per well in 24-well dishes) were inoculated with DENV, ZIKV, or HCoV-OC43 in MEM supplemented with 2% FBS, 100 IU streptomycin/penicillin per mL, 1 mM sodium pyruvate, and 10 mM HEPES (infection medium). After two hours, the inoculum was replaced with compounds in MEM supplemented with 5% FBS, 100 IU streptomycin/penicillin per mL, 1 mM sodium pyruvate, and 10 mM HEPES (maintenance medium). At the end of the experiments, supernatants were analyzed for viral yield by plaque assay as described [13]. Briefly, Vero cells were inoculated with 1:10 serial dilutions of supernatants. After two hours, the inoculum was retired and replaced with 800 μL MEM containing 1.3% methylcellulose (0.5% methylcellulose for HCoV-OC43), 2% FBS, and 10 mM HEPES. After 5 days (ZIKV) or 7 days (DENV and OC43 coronavirus) at 37 °C and 5% CO_2_, cells were washed with PBS, fixed with 2% formaldehyde, and stained using 0.5% crystal violet in 25% methanol. Infectious virus titer (pfu/mL) was determined by the number of plaques × dilution factor × (1/inoculum volume).

For SARS-CoV-2, 1.5 × 10^4^ VeroE6 cells per well were plated in a 96-well plate. The next day, the medium was replaced with 50 μL of SARS-CoV-2 infection medium (MEM supplemented with 5% FBS, 100 IU streptomycin/penicillin per mL, 10 mM HEPES, 1X NEA, 1X Glutamax, and 1X sodium pyruvate). The cells were treated with compound in 50 µL using the infection medium, immediately transferred to the BSL-3 facility, and inoculated with 50 µL infection medium containing SARS-CoV-2 at MOI = 0.01 for 72 hpi. The cells were fixed with 4% PFA for 30 min, processed for immunofluorescence, and the percentage of infected cells was calculated using the BioTek Cytation One imaging reader (Winooski, VT, USA) and the Gen5 software version 3.08 (BioTek, Winooski, VT, USA). The percentage of infected cells and total number of cells for each treatment was plotted into GraphPad Prism to perform a non-linear regression analysis, generate infectious dose–response curves, and to calculate the EC_50_ and CC_50_ values.

For influenza A virus (IAV), 2 × 10^4^ Huh7 cells per well were plated in a 96-well plate. The next day, the cells were washed twice with PBS and inoculated for two hours with IAV (MOI 0.2) in the IAV infection medium (DMEM supplemented with 0.2% BSA, 0.5 ug/mL of TPCK-treated trypsin, 100 IU streptomycin/penicillin per mL, 1X Glutamax, 10 mM HEPES). After absorption, the inoculum was removed, and cells were compound treated in the IAV infection medium. At 48 hpt, cell viability was assayed using MTS to analyze CPE induced by IAV. Typically, 100% CPE was observed in DMSO samples after 48 hpi. All the values were normalized vs. DMSO treated cells (100% CPE) and non-infected cells (0% CPE) and expressed as IAV-induced CPE.

Further information on virus assays in the Supplementary Materials can be found in the Supplementary Materials and Methods.

### 2.4. Analysis of T-1105 Conversion and Endogenous Levels of Nucleotides and PRPP

Huh7 cells were plated in 6-well plates at 4 × 10^5^ per well, two wells were used per treatment group. The next day, cells were inoculated with DENV at MOI of 0.05 in the infection medium. After two hours, inoculum was replaced by the maintenance medium containing different treatments. Supernatants and cells were collected after 12, 24, 36, and 48 hpt. Viral titers were measured from supernatants by plaque assay to corroborate the antiviral properties of the combination treatment. Cells were fixed and prepared for a liquid chromatography-tandem mass spectrometry (LC-MS/MS) analysis of T-1105-RMP, -RDP, -RTP forms, endogenous nucleotides, and PRPP. Infected cells were washed twice with cold PBS and detached using a scraper and 1 mL of cold PBS. One hundred µL (10% of the cell suspension) was stored for protein quantitation. Cell suspensions were pelleted (1500 RPM, 5 min, and 4 °C) and washed once with cold PBS. Cell pellets were resuspended in 500 µL of 60% methanol (−20 °C) and incubated at −20 °C for 18 h. The next day, samples were vortexed, heated at 95 °C for 3 min, and centrifuged at 16,000× *g* for 5 min. The supernatants were transferred to a new glass vial and dried using a SpeedVac Concentrator (Thermo Fisher Scientific, Inc., Waltham, MA, USA) at maximum pressure. Dried samples were frozen at −80 °C until the time of analysis [29].

Each dried sample was reconstituted in 200 µL of water followed by centrifugation at 14,000 rpm for 5 min (4 °C). A 50 µL aliquot of supernatant was transferred into a microtube containing 50 µL of water. The samples were then vortexed and submitted for analysis following a published LC/MS/MS method [30] with minor modifications. The LC/MS/MS system consists of an AB Sciex QTrap 5500 mass spectrometer (AB Sciex LLC, Toronto, ON, CA)and an Agilent 1260 Infinity HPLC (Agilent Technologies Santa Clara, CA, USA). The chromatographic separation of analytes was achieved on a Thermo Scientific Hypercarb column (100 × 3 mm, 5 µm). The two eluents were: (A) 0.5% diethylamine in water, pH adjusted to 10 with acetic acid; and (B) 50% acetonitrile in water. The mobile phase was delivered at a flow rate of 0.5 mL/min using a gradient of A and B as follows: 0–20 min, 5–25% B (*v*/*v*); 20–28 min, 25–50% B (*v*/*v*); 28–28.5 min, 50–95% B (*v*/*v*); 28.5–30.5 min, 95–95% B (*v*/*v*); 30.5–31 min, 95–5% B, (*v*/*v*); 31–39 min, 5–5% B (*v*/*v*). MS/MS detection of the analytes was conducted using an electrospray ion source under the fast polarity switching mode. The ion-spray voltages were set at –4500/4500 V, and the temperature at 650 °C. The curtain gas was set at 25 psi. The nebulizer gas (GS1) and turbo gas (GS2) were both set at 50 psi. The levels of mono-, di-, and triphosphate forms of T-1105 and T-1106 were adjusted vs. the total amount of protein from each sample.

### 2.5. Viral RNA Isolation for Next-Generation Sequencing

Huh7 cells were plated in 15 cm plates at 1 × 10^7^ cells per plate. Cells were inoculated with DENV at an MOI of 0.05. Two hours later, inoculum was removed and cells were treated with: DMSO (3 plates), 0.1 µM 6MMPr (3 plates), 52.5 µM T-1105 (3 plate), 0.1 µM 6MMPr/52.5 µM T-1105 (11 plates), 150 µM T-1106 (3 plates), and 0.1 µM 6MMPr/ 150 µM T-1106 (3 plates). After 72 h, supernatants were spun at 3000× *g* (4 °C) and processed for purification of viral particles (45 mL per condition, 170 mL for T-1105/6MMPr combination). Supernatants were filtered through 0.22 µm filter units (Millex, Duluth, GA, USA). Viral particles were concentrated in the interphase between 22 and 70% sucrose after two hours ultracentrifugation at 40,000 RPM (4 °C) in a 50.2 Ti fixed angle rotor. The interphase between the 22% and the 70% was recovered and diluted in 10 mM Tris pH 7.4 up to 20 mL. The virus prep was washed and concentrated to 5 mL using PES membrane concentrator tubes (100 K MWCO, 5–20 mL, Pierce, Appleton, WI, USA). Five mL of concentrated virus was diluted in 15 mL of Tris buffer for a second wash and concentration to 1 mL. Finally, the 1 mL was diluted in 10 mL of Tris buffer and concentrated to 200–500 µL. Viral RNA was extracted using the Direct-zol Kit RNA miniprep (Zymo research, Irvine, CA, USA) using 3 volumes of Trizol-LS (Thermo Scientific, Waltham, MA, USA).

### 2.6. Next-Generation Sequencing by Click Chemistry

Next-generation sequencing (NGS) and bioinformatics were performed by CliqSeq Technologies (Galveston, TX, USA). CliqSeq libraries were generated per standard Click-Seq method [31,32,33,34]. The RNA template (250 ng) was used in reverse transcription reaction with 1:35 Azido-NTPs:dNTPs ratio and SuperScript III reverse transcriptase (Invitrogen, Waltham, MA, USA). The equal molar of each indexed library was pooled and run on a Nextseq 550 system (1 × 150 reads) (Illumina, San Diego, CA, USA). Sequencing data were subjected to the usual company’s bioinformatics pipeline. Briefly, the Illumina sequencing adapter sequence 5′-AGATCGGAAGAGC-3′ was trimmed with cutadapt [35], then, the FASTX toolkit (http://hannonlab.cshl.edu/fastx toolkit/index.html/, accessed on 23 July 2020) was used to remove the remaining random nucleotides from the Illumina adapter sequence and random base-pairing as a result of azide-alkyne cycloaddition from cDNA fragments. A further quality filter was applied to remove any reads that contained more than 4% nucleotides with a PHRED score < 20. The remaining reads were aligned to DENV NGC genome (Accession Number AF038403.1) using Bowtie (v1.0.1) [36]. Point mutations in the samples comparing the DENV NGC reference were corrected using the pilon package. Furthermore, a script was written to count all nucleotide substitutions at every site in the viral genome generating an Excel file per sample. The Excel file indicates the reference genome name, the nucleotide position, the expected nucleotide in that position, the number of reads at that site (coverage), the number of mismatching As, Ts (Us), Gs, Cs, and the overall error-rate at that site (expressed in a 0 to 1 range). The Excel files were compiled in a single master file (see Supplementary Materials) and sorted by overall error-rate. Twenty point mutations common for all the samples with an error-rate higher than 0.1 were detected and excluded for further analysis. Then, all the data (error-rate < 0.1) were classified by nucleotides A, C, G, and U. We quantified individual transitions across the genome A-G, C-U, G-A, and U-C per sample by adding all the corresponding mismatches. Next, we divided the total of A-G, C-U, G-A, and U-C mismatches between the total number of readings for A, C, G, and U and multiplied for 10,000 in order to express the error-rate as errors per 10,000 nt. Additionally, the total transversions per 10,000 nt were calculated. Individual A-G, C-U, G-A, and U-C transition frequencies across the genome were calculated by dividing the number for specific mismatches between the number of readings for the indicated position. Plots and histograms depicting the distribution and frequency of A-G, C-U, G-A, and U-C error-rates across the genome were generated using GraphPad Prism 8.

### 2.7. Data Analysis

**Figure 1:**
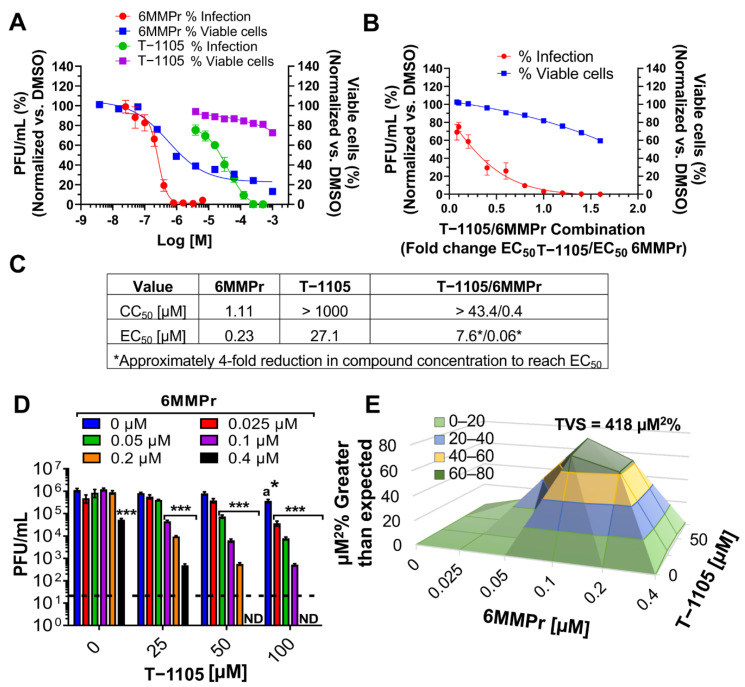
6MMPr increases T-1105 antiviral activity against DENV in Huh7 cells. (**A**) Dose-response curves for reduction of viral titers (red and green lines) and cell viability (blue and purple lines) induced by single treatments of 6MMPr and T-1105. (**B**) Dose-response curves for reduction of viral titers (red line) and cell viability (blue line) induced by T-1105/6MMPr co-treatment. X axis indicates the different combinations used. The doses for 6MMPr and T-1105 for each combination treatment were 0.08, 0.1, 0.2, 0.4, 0.6, 0.8, 1, 1.2, 1.4, and 1.6 times their respective EC_50_. Data are from three biological replicates. (**C**) CC_50_ and EC_50_ values for 6MMPr, T-1105, and the combination. (**D**) The bar graph depicts the reduction of viral titer promoted by T-1105/6MMPr co-treatment. X axis represents four different concentrations of T-1105 and the bar color indicates the concentration of 6MMPr. Mean ± SEM of viral titers (PFU/mL) from two independent experiments by duplicates is plotted. (**E**) 3D plot indicating synergistic antiviral effect. “a”, indicates significant antiviral activity induced by T-1105 alone; *, *p* < 0.005; ***, *p* < 0.001; ND, none detected; black dotted line, limit of detection. TVS = total volume of synergy calculated at 99.9% confidence interval.

**Figure 2:**
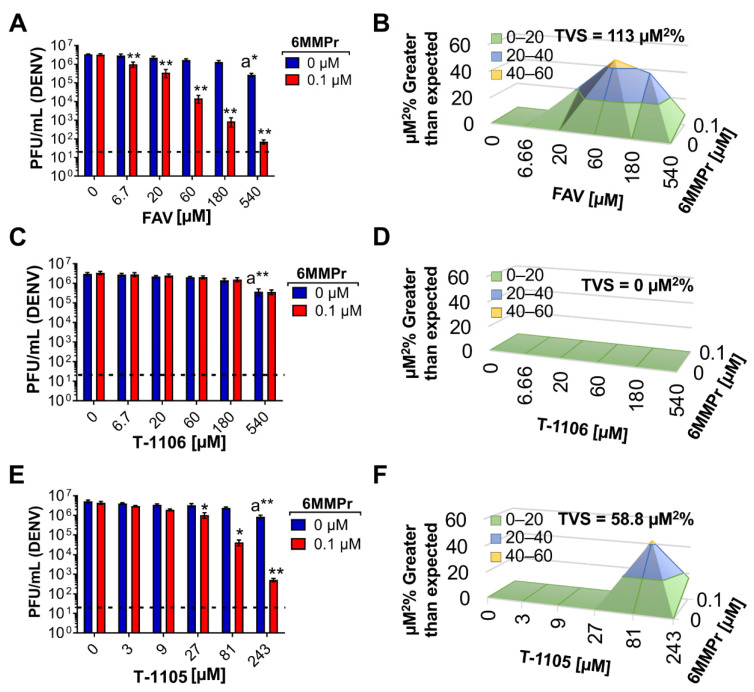
6MMPr potentiates antiviral effect of nucleobase but not nucleoside in DENV-infected Huh7 cells. The bar graphs depict the reduction of viral titer promoted by FAV/6MMPr (**A**), T-1106/6MMPr (**C**), and T-1105/6MMPr (**E**) co-treatments. Mean ± SEM of viral titers (PFU/mL) from two independent experiments conducted in duplicate are plotted. “a” indicates significant antiviral activity induced by the nucleobase or the nucleoside alone. * *p* < 0.05; ** *p* < 0.005; Black dotted line indicates limit of detection. (**B**,**D**,**F**) show the 3D plots indicating synergistic antiviral effect. Total volume of synergy = TVS. All TVS calculated at 99.9% confidence interval.

**Figure 3:**
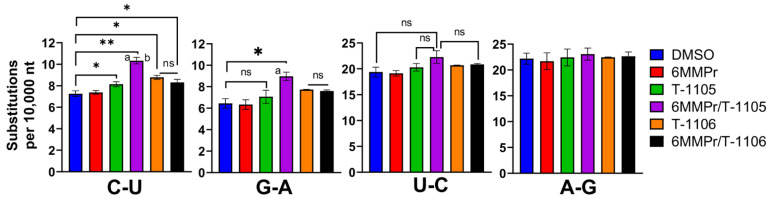
6MMPr increases the C-U and G-A transition frequencies observed in DENV-infected cells treated with T-1105 but not T-1106. Huh7 cells inoculated with DENV (MOI 0.05) were treated with DMSO, 0.1 µM 6MMPr, 52.5 µM T-1105, T-1105/6MMPr combination, 150 µM T-1106, and T-1106/6MMPr combination for 72 hpi. Viral particles from supernatants were concentrated, viral RNA prepared, and the number of substitutions analyzed by NGS. The graphs represent the mean ± SD of the total number of transition (C-U, G-A, U-C, and A-G) identified per 10,000 nt. *, *p* < 0.05; **, *p* < 0.005; ns, non-significant; “a”, *p* < 0.05 compared to T-1105 alone; “b”, *p* < 0.05 compared to T-1106/6MMPr.

**Figure 4:**
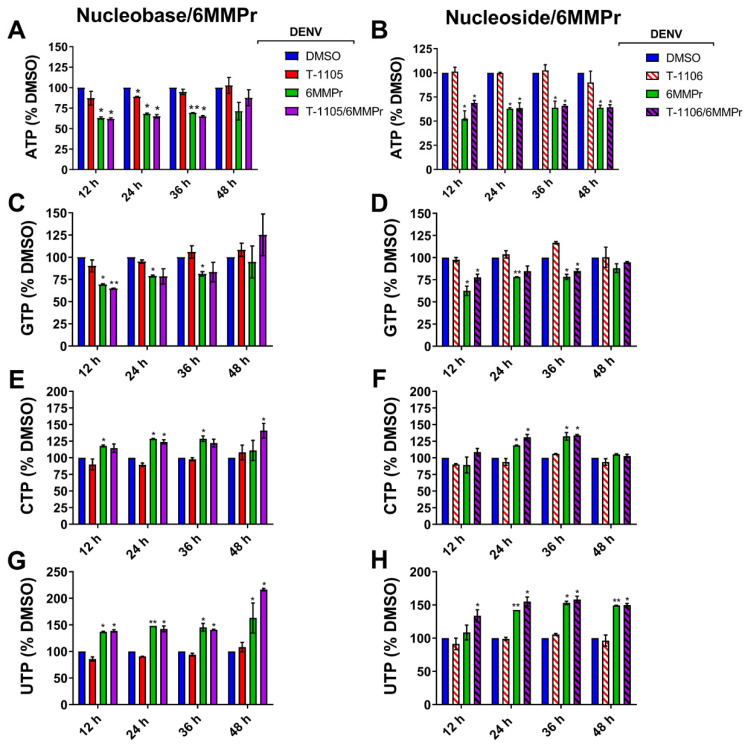
6MMPr reduces the endogenous levels of purine nucleotides (ATP and GTP) and increases the levels of pyrimidine nucleotides (CTP and UTP). Endogenous ATP (**A**,**B**), GTP (**C**,**D**), CTP (**E**,**F**), and UTP (**G**,**H**) were analyzed by LC/MS/MS. Nucleotide levels were normalized vs. DMSO-treated cells for each time point and expressed as % DMSO. Endogenous nucleotide levels were adjusted vs. the total amount of protein from each sample. The mean ± SD of two independent experiments were used to build the graphs. *, *p* < 0.05; **, *p* < 0.005.

**Figure 5:**
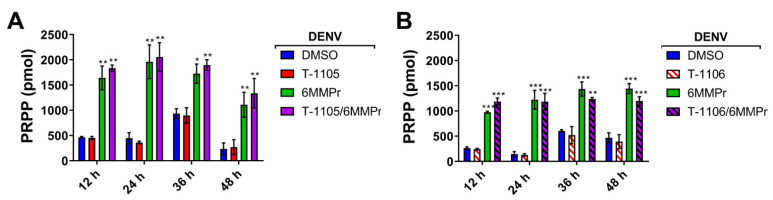
6MMPr induces accumulation of PRPP. Endogenous levels of PRPP were quantified by LC/MS/MS and expressed as total picomoles (pmol) for samples for both nucleobase (T-1105) (**A**) and nucleoside (T-1106) (**B**) experiments. PRPP levels were adjusted vs. the total amount of protein from each sample. The mean ± SD of two independent experiments were used to build the graphs. *, *p* < 0.05; **, *p* < 0.005; ***, *p* < 0.001.

**Figure 6:**
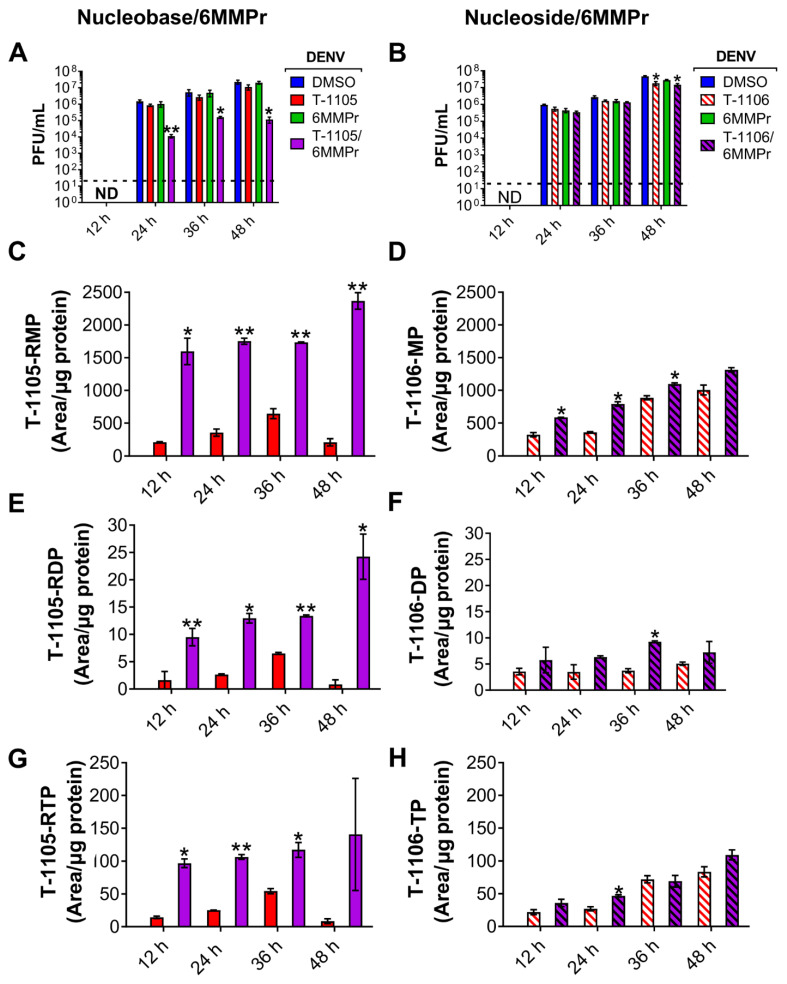
6MMPr increases the conversion to the active form of nucleobase T-1105 but not of its nucleoside analog T-1106. Huh7 cells were inoculated with DENV-2 and treated with DMSO, the nucleobase T-1105 (52.5 µM), 6MMPr (0.1 µM), and the T-1105/6MMPr co-treatment. After 12, 24, 36, and 48 hpt, supernatants were analyzed for viral titers (expressed as PFU/mL in (**A**) and cells were fixed and prepared for LC/MS/MS analysis of T-1105 ribonucleoside-5’-monophosphate (T-1105-RMP in (**C**)), T-1105 ribonucleoside-5´-diphosphate (T-1105-RDP in (**E**)), and T-1105 ribonucleoside-5´-triphosphate (T-1105 RTP in (**G**)). Similar assay was performed for the nucleoside T-1106 (150 µM) and co-treatment with 6MMPr. Graph in (**B**) indicates the viral titers. (**D**,**F**,**H**) correspond to the T-1106 5´ monophosphate (T-1106-MP), the T-1106 5´ diphosphate (T-1106-DP), and the T-1106 5´ triphosphate (T-1106-TP) forms, respectively. Mono-, di-, and tri-phosphate levels are expressed as the peak area normalized vs. the amount of total protein of each sample (area/µg protein). The plots represent the mean ± SEM of two independent experiments. * *p* ≤ 0.05; ** *p* ≤ 0.005. ND, none detected; black dotted line, limit of detection.

**Figure 7:**
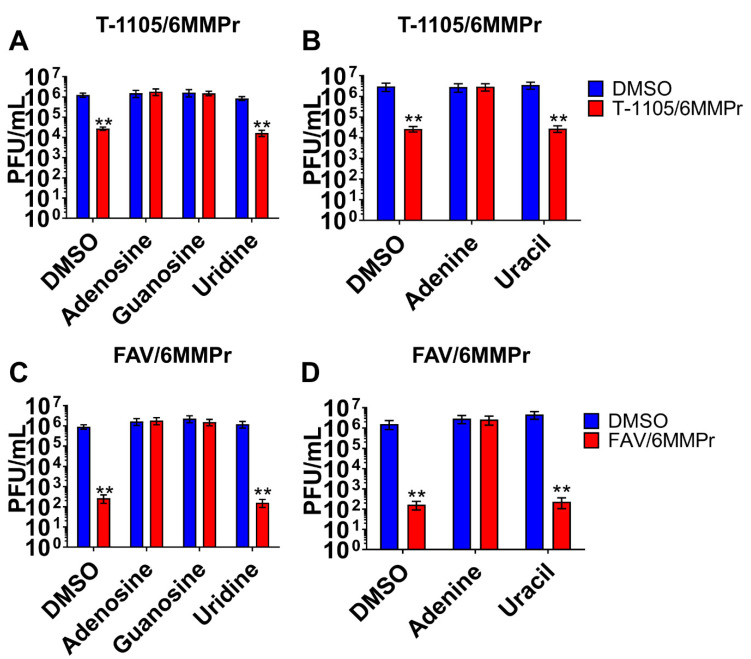
Adenosine, adenine, and guanosine block the antiviral effect induced by nucleobase/6MMPr combinations. DENV-inoculated Huh7 cells treated with DMSO, T-1105/6MMPr (52.5 µM/0.1 µM) (**A**,**B**), or favipiravir/6MMPr (275 µM/0.1 µM) (**C**,**D**) in the absence or presence of exogenous nucleosides (**A**,**C**) or nucleobases (**B**,**D**). The plots represent the mean ± SEM of viral titers expressed as PFU/mL from two independent experiments with samples performed in triplicate. ** *p* ≤ 0.005.

**Figure 8:**
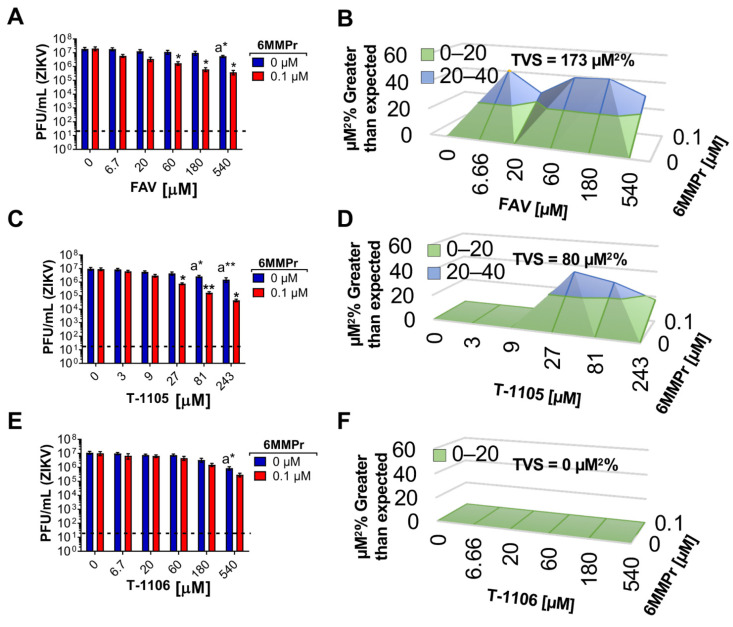
6MMPr enhances antiviral effect of nucleobase but not nucleoside in ZIKV-infected cells. Bar graphs represent the viral titer reduction induced by favipiravir (FAV)/6MMPr (**A**), T-1105/6MMPr (**C**), and T-1106/6MMPr (**E**) co-treatments in Huh7 cells inoculated with wild type ZIKV. Mean ± SEM from two independent experiments conducted in duplicate are plotted. “a” indicates significant antiviral activity induced by the nucleobase or the nucleoside alone. * *p* < 0.05; ** *p* < 0.005. Black dotted line indicates limit of detection. (**B**,**D**,**F**) show the 3D plots indicating synergistic or additive antiviral effect. Total volume of synergy = TVS. All TVS calculated at 99.9% confidence interval.

**Figure 9:**
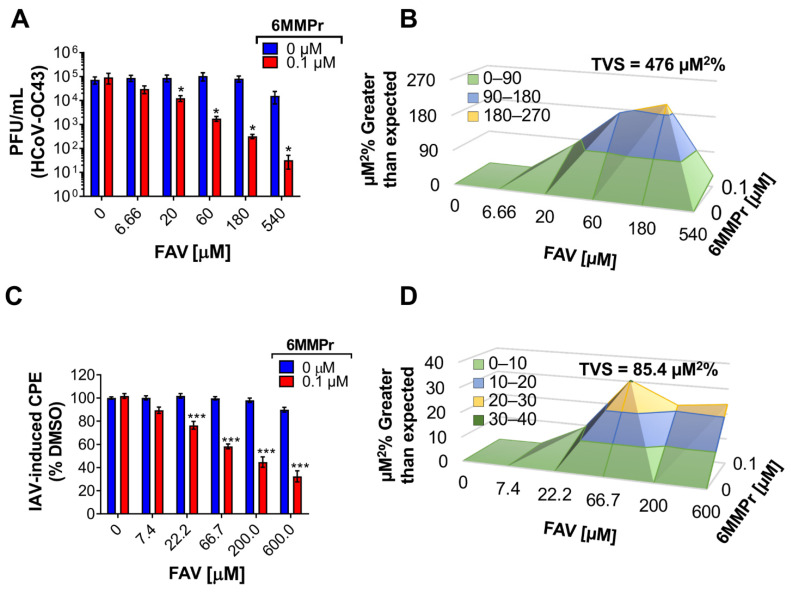
6MMPr increases the favipiravir antiviral potency against IAV (H1N1) and HCoV-OC43. (**A**) HCoV-OC43 titer of supernatants collected 72 hpi from Huh7 cells inoculated at MOI of 0.1 and treated with different concentrations of FAV in combination with 6MMPr or DMSO. Bars depict the mean ± SEM from two independent experiments conducted in duplicate. (**B**) The 3D plot indicating synergistic antiviral effect. (**C**) Cytopathic effects (CPE) induced by IAV in Huh7 cells inoculated at MOI of 0.2 and treated with increasing concentrations of favipiravir (FAV) in combination with 0.1 µM 6MMPr (red bars) or DMSO (0 µM, blue bars) for 48 hpi. The bars represent mean CPE normalized vs. DMSO-treated cells ± SEM of three independent experiments. *, *p* < 0.05; ***, *p* < 0.001. (**D**) The 3D plot indicating synergistic antiviral effect. Total volume of synergy = TVS, calculated at 99.9% confidence interval.

Synergy was analyzed with the software MacSynergy II™ [37]. For a synergy analysis, MacSynergy II™ [37] graphically plots 3D drug interactions above (synergy) or below (antagonism) a neutral surface (additive). The software also generates a value referred to as the volume of synergy (excess activity compared to the predicted additive effect) indicated in the Y-axis of the 3D plot. Values between 25 and 50 µM^2^% indicate minor but significant synergy, values between 50 and 100 µM^2^% indicate moderate synergy, and values >100 µM^2^% indicate strong synergy. Negative values, below the additive plane, equal antagonistic results. All values were generated using the 99.9% confidence interval. Plot construction and the calculation of CC_50_, EC_50,_ and TCID_50_ values were developed in GraphPad Prism 8. Differences between treatments and control groups were evaluated using the GraphPad Prism 8 and SigmaPlot/Stat package 12. For Figures 1D, 2, 4, 5, 8, and 9A a two-way ANOVA with Holm–Sidak’s multiple comparison was applied. For Figures 3, 6, 7, and 9C, parametric or nonparametric tests and the appropriate post-hoc test were applied. If data did not meet the assumptions of normality (Shapiro–Wilk test) and equal variance test, then multiple Mann–Whitney U tests were performed.

## 3. Results

### 3.1. 6MMPr in Combination with T-1105 or Favipiravir Results in Synergistic Increases in Antiviral Activity against Dengue Virus (DENV)

To find potential new antimetabolites that enhance the antiviral activity of nucleobases, we co-treated a DENV luciferase replicon cell line with 8 µM T-1105 and a small panel of known antimetabolites (Figure S1) at non-toxic concentrations (Table S1). We used T-1105 because it is a more potent inhibitor against DENV when compared to favipiravir [13]. We identified the purine derivative and phosphoribosyl pyrophosphate amidotransferase (PPAT) inhibitor 6MMPr as a possible enhancer of T-1105 antiviral activity (Figure S1). PPAT catalyzes the first step in de novo purine nucleotide synthesis such that inhibition of PPAT should result in a reduction in purine NTP biosynthesis and cause a buildup of PRPP, a molecule critical for the bioconversion of nucleobases to RMPs [22]. Ribavirin was also identified as a T-1105 enhancer (Figure S1) similar to a published report of ribavirin enhancing the antiviral activity of favipiravir [26]. Going forward, we focused on 6MMPr because it has not previously been described in antiviral combinations.

We determined EC_50_ and CC_50_ values for individual 6MMPr and T-1105 treatments (Figure 1A−E). Huh7 cells were inoculated with DENV2 and treated with increasing concentrations of either 6MMPr (0–0.4 µM) or T-1105 (0–1 mM). At 72 h post-treatment (hpt), supernatants were analyzed for infectious particles by plaque assay and that data were used to determine EC_50_ values for each compound. In parallel, the viability of the compound-treated non-infected cells was determined to assign CC_50_ values for each compound (Figure 1A,C). Both 6MMPr and T-1105 inhibited viral replication in a concentration-dependent manner resulting in a 0.23 µM EC_50_ for 6MMPr and 27 µM EC_50_ for T-1105 (similar to EC_50_ reported previously [13]). In addition, EC_50_ values were determined for the combination treatment (Figure 1B). The dose for each compound in the combination treatment was based upon their respective EC_50_ values (0.23 µM for 6MMPr and 27 µM for T-1105). The final concentration for each compound in the combination was 0, 0.08, 0.1, 0.2, 0.4, 0.6, 0.8, 1, 1.2, 1.4, or 1.6 times their respective EC_50_ values. The combination with a 50% reduction in viral titer was 7.6 µM T-1105/0.06 µM 6MMPr, a four-fold reduction in compound concentration compared to their EC_50_ values when used alone with no effect on cell viability (Figure 1B,C).

We next asked if the inhibitory effects of the T-1105/6MMPr combination were synergistic. Huh7 cells were inoculated with DENV2 and treated with increasing concentrations of 6MMPr (0–0.4 µM) in combination with different concentrations of T-1105 (0, 25, 50, and 100 µM). After 72 h, viral titers were measured in the supernatants. Approximately 0.5–1-log reduction in infectious virus was observed when either 6MMPr or T-1105 alone was added to cultures at the highest concentration (0.4 μM or 100 μM, respectively) (Figure 1D). Importantly, T-1105/6MMPr combinations considerably increased the reduction in infectious virus produced with no infectious virus detected from cultures treated with 50 or 100 μM T-1105 combined with 0.4 μM 6MMPr (Figure 1D). Further analyses indicated a strong synergistic effect with an accumulated synergy volume of 418 µM^2^% at 99.9% CI (for more information on synergy calculations via MacSynergy II, volume of synergy and range of values yielding moderate or strong synergy, see Materials and Methods). The combination with the highest synergy was 50 µM T-1105/0.1 µM 6MMPr (Figure 1E). Based upon the results with T-1105, a scaled-down experiment with favipiravir and 6MMPr showed a four-log reduction in infectious virus production and strong synergy (113 µM^2^% at 99.9% CI) when 0.1 μM 6MMPr was combined with the highest favipiravir concentration (540 µM) (Figure 2A,B). Interestingly, the T-1106/6MMPr combination did not increase the reduction in infectious virus production over T-1106 alone and no synergy was observed (Figure 2C,D). In this scaled-down format, T-1105 showed synergistic inhibition when combined with 0.1 μM 6MMPr as expected (Figure 2E,F). These results demonstrate that 6MMPr synergistically increased the antiviral efficacy of nucleobases T-1105 and favipiravir and this effect was not observed for the nucleoside T-1106.

### 3.2. 6MMPr Co-Treatment Enhanced the Viral Mutation Frequency Observed with T-1105 but Not T-1106

Favipiravir and T-1105 increase the frequency of transition mutations for RNA viruses [11,13,38,39,40] and this effect drives the lethal mutagenesis that is at least partly responsible for their antiviral effects. We wanted to determine if the enhanced antiviral effect with nucleobase/6MMPr combinations coincided with an enhanced mutation frequency. We focused on T-1105 and DENV because we have examined mutagenesis with them in a previous study [13] and the availability of the nucleoside form of T-1105, T-1106. T-1106 was used as a control because treatment also increases the mutation frequency of DENV [13] but 6MMPr does not synergize the T-1106 antiviral effect and would not be expected to affect mutation frequency.

Huh7 cells were inoculated with DENV (MOI = 0.05) and treated with DMSO, 0.1 µM 6MMPr, 52.5 µM T-1105, T-1105/6MMPr combination, 150 µM T-1106, and T-1106/6MMPr combination. We chose the concentrations 52.5 µM T-1105 with 0.1 µM 6MMPr because they induced a high anti-DENV synergistic effect (Figure 2) and the supernatants still contained enough viral particles to analyze the genome sequence. T-1105 at 52.5 µM corresponds to 2.5 times the EC_50_ previously reported (21 µM) so we used 150 µM T-1106, equivalent to 2.5 times the EC_50_ value previously reported (60 µM) [13]. At seventy-two hpt, we purified virus-associated RNA from the supernatants and used next-generation sequencing (NGS) to analyze the sequence of virus-associated genomes for all treatment groups. The number of transition mutations (C-U, G-A, U-C, and A-G) was calculated for all the treatments and expressed as substitutions per 10,000 nucleotides (Figure 3). The C-U and G-A substitutions were significantly increased for T-1105/6MMPr-treated cultures when compared to T-1105 alone. The 6MMPr co-treatment did not significantly increase any transition mutation frequency for T-1106-treated cultures compared to T-1106 alone (Figure 3).

This result is also evident when the data were presented in histograms with the transition C-U and G-A frequency peak shifted to the right for T-1105/6MMPr compared to T-1105 alone (Figure S2A,C). Additionally, the analysis of individual C-U and G-A transition frequencies across the genome indicated that the augmented mismatch frequency observed during the T-1105/6MMPr combination was caused by multiple, homogenous increases as predicted for lethal mutagenesis and not by single outliers (Figure S2B,D). No differences were detected in the number or frequency of U-C and A-G transitions or for any transversions regardless of treatment (Figure 3, and Figures S2E–H, and S3). The increased transition mutation frequency occurred for the T-1105/6MMPr combination but not the T-1106/6MMPr combination, closely paralleling the antiviral synergy results and suggesting an increase in mutation frequency contributes to the synergy.

### 3.3. 6MMPr Increases Intracellular Levels of T-1105-RMP, -RDP, -RTP, and PRPP While Reducing Endogenous ATP and GTP Levels

PPAT inhibition by 6MMPr results in an accumulation of PRPP as well as a reduction in cellular purine NTP biosynthesis [41,42]. We examined the effect of the 6MMPr treatment on the levels of PRPP and NTPs in DENV-infected Huh7 cells. The 6MMPr treatment alone or in combination with T-1105 or T-1106 (Figure 4) reduced the levels of endogenous purine NTPs in DENV-infected cells compared to cultures treated with DMSO or T-1105 or T-1106 alone. Increases in the pyrimidine TPs CTP and UTP were observed during 6MMPr treatments in combination or alone (Figure 4), consistent with a previous report [43]. Finally, the 6MMPr treatment alone or in combination increased the accumulation of endogenous PRPP at all timepoints (Figure 5).

Phosphoribosyltransferases use PRPP to convert nucleobases to their RMP form [22]. We hypothesized that the PRPP accumulation induced by 6MMPr-mediated PPAT inhibition could increase the conversion of the nucleobase T-1105 to its RMP, RDP, and RTP forms. We focused on T-1105 in this analysis because of the availability of the corresponding nucleoside T-1106 as a measure of specificity of any effect. To test the hypothesis, DENV-inoculated cells were treated with DMSO, 0.1 µM 6MMPr, 52.5 µM T-1105, 150 µM T-1106, and the corresponding combinations. Cell supernatants and lysates were collected at 12, 24, 36, and 48 hpt. The lysates were analyzed for levels of T-1105-RMP, -RDP, and -RTP forms (Figure 6A−H). The 6MMPr co-treatment increased the intracellular levels of T-1105-RMP, -RDP, and -RTP at all timepoints compared to the T-1105-treated cells (Figure 6C,E,G). The 6MMPr-dependent enhancement effect was not observed for the nucleoside T-1106 (Figure 6D,F,H) as expected likely due to the nucleoside’s different pathway to the MP form compared to the nucleobase. The viral titer of the collected supernatants was measured to corroborate the synergistic reduction of viral titer at each time point for the combination treatment compared to either treatment alone (Figure 6A,B). The observed increase in T-1105 potency occurred concomitantly with the increase in T-1105-RMP, -RDP, and -RTP forms and reduction in purine NTP levels.

### 3.4. Supplementation with Exogenous Purines Abolished the Nucleobase/6MMPr Antiviral Activity

Based on our previous observations, we predicted that supplementation with exogenous purines would reduce the antiviral potency of the T-1105/6MMPr combination by reestablishing the endogenous NTP levels, competing with cellular enzymes required for the increase in the T-1105 conversion, and blocking the incorporation of T-1105-RTP into the viral genome. DENV-inoculated cells were treated with 52.5 µM T-1105/0.1 µM 6MMPr or DMSO as control, in the presence or absence of an exogenous nucleoside (adenosine, guanosine, or uridine) or nucleobase (adenine or uracil) at 200 µM. At 72 hpt, supernatants were analyzed for infectious particle production by plaque assay (Figure 7). As expected, the T-1105/6MMPr co-treatment reduced the number of plaque-forming units by up to 1.6 logs compared to control cells. Adenosine, guanosine, and the nucleobase adenine efficiently blocked the antiviral effect. Additionally, the exogenous supplementation of pyrimidines, uridine, and uracil, did not block the 6MMPr/T-1105 antiviral effect (Figure 7A,B). The exogenous purine supplementation also blocked the antiviral properties observed when 275 µM favipiravir (2.5 times the reported EC_50_) was combined with 0.1 µM 6MMPr (Figure 7C,D).

### 3.5. 6MMPr Synergizes the Antiviral Effect of T-1105 and Favipiravir against Zika Virus and Respiratory RNA Viruses including SARS-CoV-2

We were interested in examining other RNA viruses for possible synergistic inhibition and initially chose another flavivirus, Zika virus (ZIKV). The addition of 0.1 µM 6MMPr increased the anti-ZIKV effect of favipiravir (strong synergy) (Figure 8A,B) and T-1105 (moderate synergy) (Figure 8C,D), but not T-1106 (Figure 8E,F) in a titer reduction assay performed in Huh7 cells. In addition, a synergistic effect was observed for a reduction in ZIKV replication in HFFs for the T-1105/6MMPr combination when a luciferase reporter virus was used (Figure S4).

Furthermore, 6MMPr increased the antiviral effect of favipiravir with a synergistic effect against respiratory viruses such as human coronavirus OC43 (HCoV-OC43) (Figure 9A,B), a cause of relatively minor upper respiratory illness [44,45], and the virus favipiravir was originally developed to treat, IAV [2,46] (Figure 9C,D). These results demonstrate that the 6MMPr-dependent enhancement of antiviral activity of nucleobases such as favipiravir occurs against a broad spectrum of RNA viruses and in a variety of cultured cells.

Favipiravir has been evaluated as a potential treatment for SARS-CoV-2 infections [6,8,47], so we examined the nucleobase/6MMPr combination for inhibition of SARS-CoV-2 in cell culture. First, we validated an immunofluorescence-based assay where cells were inoculated with different MOIs (0.01, 0.1, and 1) in the presence of DMSO, favipiravir, 6MMPr, and favipiravir/6MMPr for 48 or 72 hpi. The cells were fixed, stained with an antibody recognizing the nucleoprotein (N), and the number of N-positive cells were counted. As expected, favipiravir reduced the number of infected cells and the titer of virus produced by treated Vero E6 cells (Figure S5) [4,10,48], and 6MMPr increased the antiviral effect induced by favipiravir. In addition, the favipiravir/6MMPr co-treatment enhanced the favipiravir anti-SARS-CoV-2 effect in human lung Calu-3 cells (Figure S6).

**Figure 10:**
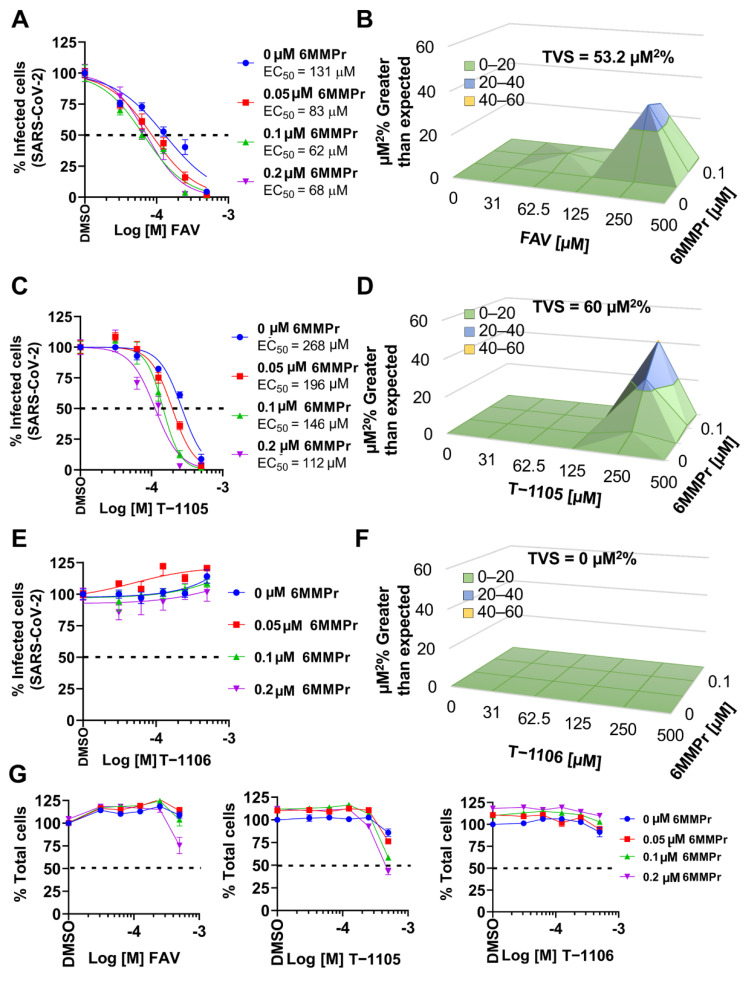
6MMPr synergistically enhances anti-SARS-CoV-2 activity of favipiravir. Vero-E6 cells were inoculated with SARS-CoV-2 at MOI of 0.01 for 72 hpi in the presence of 0, 31, 62.5, 125, 250, and 500 µM of favipiravir (FAV) (**A**), T-1105 (**C**), and T-1106 (**E**). The percent of infected cells was calculated by immunostaining of viral nucleoprotein and used to build infectious dose-response curves. EC_50_ values are indicated for favipiravir and T-1105 alone and in combination with 6MMPr. The 3D plots in (**B**,**D**,**F**) indicate synergistic antiviral effect or lack thereof for each combination. (**G**) Total number of cells quantified by DAPI staining from panel (**A**,**C**,**E**) was used to calculate % cell viability for FAV, T-1105, and T-1106 alone or in combination with 6MMPr. The graphs depict the mean ± SEM of two independent experiments with three biological replicates. Total volume of synergy = TVS. All TVS calculated at 99.9% confidence interval. Dotted black line indicates 50%.

To evaluate possible synergy, Vero-E6 cells were inoculated with SARS-CoV-2 (MOI of 0.01) and treated with different concentrations of favipiravir (0–500 µM) in combination with different concentrations of 6MMPr (Figure 10A−G). Favipiravir alone reduced the number of infected cells in a dose-dependent manner with an EC_50_ = 131 µM. Lower EC_50_ values (Figure 10A) and a modest synergy (Figure 10B) were obtained when combined with different 6MMPr combinations. Favipiravir CC_50_ values when used alone or combined with 0.05, 0.1, or 0.2 µM 6MMPr were greater than 500 µM (Figure 10G). Similarly, T-1105 inhibited SARS-CoV-2 infection (EC_50_ = 268 µM) and showed a modest synergistic effect when combined with 6MMPr (Figure 10C,D,G). T-1105 CC_50_ values in combination with 0.1 µM 6MMPr or 0.2 µM 6MMPr were approximately 500 µM. The T-1106 nucleoside did not show any antiviral effect alone or in combination with 6MMPr (Figure 10E–G).

## 4. Discussion

Here, we report a synergistic antiviral effect induced by the novel combination of the antimetabolite 6MMPr and the nucleobases T-1105 or favipiravir. This effect extends to RNA viruses from three virus families and in multiple different cell lines. We further describe a comprehensive analysis of a mechanism of action for increased combination antiviral activity stemming from an increase in viral genomic mutations. Lastly, we show that the increase in viral mutation coincides with an increase in nucleobase modification to the active antiviral RTP form by the co-treatment of the antimetabolite 6MMPr.

**Figure 11:**
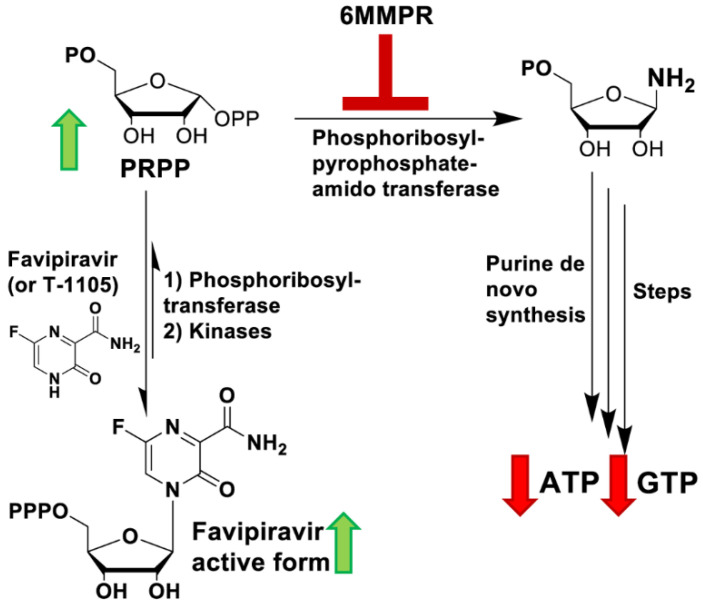
Potential mechanism for 6MMPr effects on nucleobase and purine nucleoside conversion to the relevant triphosphate forms. 6MMPr inhibits phosphoribosyl pyrophosphate amidotransferase (PPAT) resulting in an inhibition of de novo purine synthesis. PPAT inhibition causes PRPP accumulation and increases nucleobase conversion to the ribonucleoside-5′-monophosphate form by cellular phosphoribosyltransferases and kinases. Green arrows pointing upwards indicate an increased accumulation and red arrows pointing downwards a decreased accumulation.

As summarized in Figure 11, 6MMPr inhibits the committed step of de novo purine biosynthesis by blocking the PPAT-catalyzed conversion of PRPP into 5′-phosphoribosyl-1-amine producing at least two important outcomes. One is a reduction in the endogenous levels of ATP and GTP and the second is the accumulation of PRPP [21,49,50,51] (Figure 4 and Figure 5). We propose that PRPP accumulation induced by 6MMPr increases the conversion of T-1105 to T-1105-RMP by a salvage pathway phosphoribosyltransferase and ultimately results in an increase in the subsequent RDP and RTP forms. Purine nucleobases can be converted to the RMP form via phosphoribosyltransferases as part of the cellular salvage pathway [22]. A nucleobase/salvage pathway-specific effect could explain the failure of a 6MMPr co-treatment to increase T-1106 nucleoside mono-, di-, and triphosphate forms because T-1106 conversion to the monophosphate form does not occur via the salvage pathway but through kinase phosphorylation.

In general, the NTP forms of T-1105 and favipiravir can be incorporated into the nascent viral RNA with similar efficiency compared to ATP or GTP [14,52,53]. Therefore, a reduction in the endogenous levels of ATP and GTP could be beneficial for the antiviral effect exerted for these nucleobases. We hypothesize that the reduction of purine levels induced by 6MMPr could favor the use of the T-1105-RTP form by viral polymerases and increase antiviral effects. The reversal of the antiviral effect by the addition of exogenous purine nucleobase/nucleosides (Figure 7) is consistent with the importance of the relative levels of the T-1105-RTP and cellular NTPs to the antiviral effects observed in the T-1105/6MMPr combination.

Our results suggest that both the reduction in endogenous purine NTP synthesis and an increase in the active antiviral nucleobase-RTP form are required for synergistic antiviral effects mediated by 6MMPr. No synergy was observed for the 6MMPr/T-1106 co-treatment, possibly due to the lack of increased conversion of the active TP form. The reduction in endogenous purine NTP synthesis, in the absence of an increase in the T-1106-TP form for the T-1106/6MMPr co-treatment, is not sufficient to produce a synergistic effect. For the T-1105/6MMPr co-treatment, supplementation with exogenous purines abrogates enhanced antiviral activity suggesting that an increase in the active RTP form alone is unlikely to produce a synergistic antiviral effect.

Increasing evidence suggests that T-1105 and favipiravir can induce lethal mutagenesis for different RNA viruses [11,13,39,40,54,55]. In this work, we determine that the 6MMPr co-treatment builds an environment favorable for increased T-1105-RTP incorporation into the DENV genome, increasing the C-U and G-A transition mutation frequency. RNA virus replication error frequencies are finely regulated and exist close to their tolerance threshold [56,57,58] indicating that small increases in the mutation frequencies will result in significant antiviral effects [18,59].

Favipiravir has been studied in humans for influenza and emerging viruses such as Ebola virus and SARS-CoV-2 [9,47,60,61,62]. Results from these trials suggest a complex pharmacokinetics profile. Moreover, mixed results in efficacy for different viruses indicate problems to reach the half-maximal effective concentrations in treatment settings [63]. The combination strategy proposed here could help to reduce pharmacokinetic issues and possibly even reduce the favipiravir dose. Here, we report the proof-of-concept that the PPAT inhibitor 6MMPr increases the intracellular levels of the T-1105 active form and synergistically increases the antiviral activity of the nucleobases T-1105 and favipiravir against a panel of RNA viruses, including SAR-CoV-2. Although the combination strategy reported here uses non-toxic doses of 6MMPr, 6MMPr use in humans can produce side effects [64], making it unclear if 6MMPr would directly translate to clinical trials. However, this work establishes the foundation for the development of safer small molecules targeting PPAT as possible co-treatments with nucleobases like favipiravir in response to emerging RNA virus infections.

## Data Availability

The data presented in this study are available in the article and Supplementary Materials.

## Supplementary Materials

The following are available online at https://www.mdpi.com/article/10.3390/v13122508/s1, Table S1: Antimetabolite screening, Figure S1: Initial nucleobase (T-1105) activity enhancement screening, Figure S2: 6MMPr increases the C-U and G-A transition frequencies observed in DENV-infected cells treated with T-1105 but not T-1106, Figure S3: Treatments and combinations do not alter the total number of transversions, Figure S4: 6MMPr enhances anti-ZIKV effect of T-1105, Figure S5: 6MMPr enhances anti-SARS-CoV-2 activity of favipiravir, Figure S6: 6MMPr enhances anti-SARS-CoV-2 activity of favipiravir in multiple cell lines, Supplementary Materials and Methods, References, and Excel Sequencing Master File.
