# Supplementary material for: Enhancing the Antiviral Potency of Nucleobases for Potential Broad-Spectrum Antiviral Therapies
Source: Viruses. 2021 Dec 14;13(12):2508. doi: 10.3390/v13122508 (PMC8705664; doi:10.3390/v13122508)
Supplement: Supplementary file 1 [file viruses-13-02508-s001.zip › Soto-Acosta et al Supplement.pdf]

Supplementary Materials

Enhancing the antiviral potency of nucleobases for potential broad spectrum antiviral therapies

Ruben Soto-Acosta, Tiffany C. Edwards, Christine D. Dreis, Venkatramana D. Krishna, Maxim C. Cheeran, Jiashu Xie, Li Qiu, Laurent F. Bonnac*, Robert J. Geraghty*

Table of contents

Table S1. Antimetabolite screening, pg S2
Figure S1. Initial nucleobase (T-1105) activity enhancement screening, pg S3
Figure S2. 6MMPr increases the C-U and G-A transition frequencies observed in DENV infected cells treated with T-1105 but not T-1106, pg S4
Figure S3. Treatments and combinations do not alter the total number of transversions, pg S5
Figure S4. 6MMPr enhances anti-ZIKV effect of T-1105, pg S6
Figure S6. 6MMPr enhances anti-SARS-CoV-2 activity of favipiravir, pg S7
Figure S7. 6MMPr enhances anti-SARS-CoV-2 activity of favipiravir in multiple cell lines, pg S8
Supplemental Materials and Methods, pgs S9-S10
References, pg S11

Antimetabolite	[µM]	DENV replicon	
		% of inhibition	% Viability reduction
6-mercaptopurine.H2O (6-MP)	0.1	0	16
	0.25	0	13
	0.5	21	4.2
	5	87	75
Raltitrexed	0.01	0	0
	0.025	17	14
	0.05	0	41
Naphtoquinone	0.05	0	11
	0.1	0	4.5
	5	100	100
Lometrexol	0.01	0	3.9
Aminopterin	0.005	15	5.7
	0.01	82	85
	0.05	84	81
	1	86	84
	10	84	85
6-methylmercaptopurine riboside (6MMPr)	0.025	0	20
	0.05	5.9	5.9
	0.1	15	1.1
	0.2	3.7	0
	0.25	17	4.8
	0.5	37	13
	1	99	43
	10	99	88
5-fluorouracil	0.2	0	0
	0.5	9.2	15
	0.5	19	13
	1	45	38
	10	88	85
Brequinar	2.5	2.4	17
	2.5	3.6	5.2
	10	79	54
Leflunomide	5	18	17
	10	29	2.4
6-azauridine	1	1	5
	5	87	1.3
	10	97	28
3-deazauridine	1	0	0
	2	0	8
	2.5	5.7	16
	5	8.2	24
	10	24	31
Mizoribine	1	7.7	112.8
Ribavirin	1	38	3.7
	5	77	0
	10	87	17

Table S1. Antimetabolite screening. Antimetabolites were tested at different concentrations in the BHK-DENV replicon system for antiviral activity (Luciferase) and cell toxicity (MTS). Relative Luciferase signal (RLU) and MTS 490 absorbance were normalized vs DMSO treated cells (0%) and expressed as % inhibition and % viability reduction, respectively.

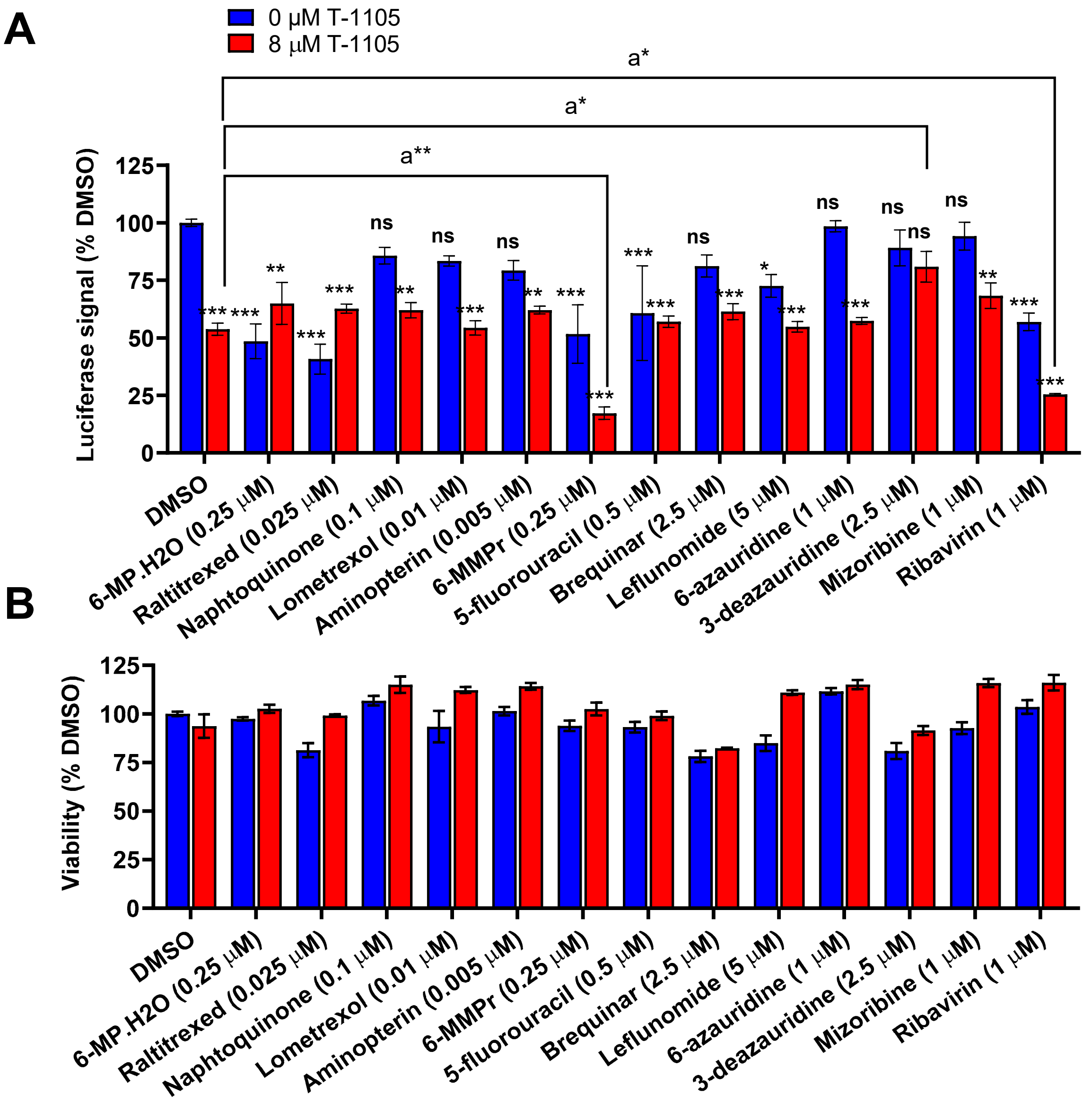

Figure S1. Initial nucleobase (T-1105) activity enhancement screening. DENV replicon cells were treated with different known antimetabolites at the concentrations indicated in presence or absence of 8 µM T-1105. After 72 hrs, the cells were analyzed for luciferase signal as a measure of DENV replicon replication (**A**). The graph represents the normalized mean ± SEM of luciferase signal from samples in triplicate. "a" indicates comparison vs 8 µM T-1105-DMSO *, P<0.05; **, P<0.005; ***, P<0.001; "ns" indicates non-significant when compared with 0 µM T-1105-DMSO. In parallel, cell viability was measured by MTS assay (**B**). The graph indicates the mean ± SEM of 490 nm absorbance normalized vs DMSO of triplicate samples.

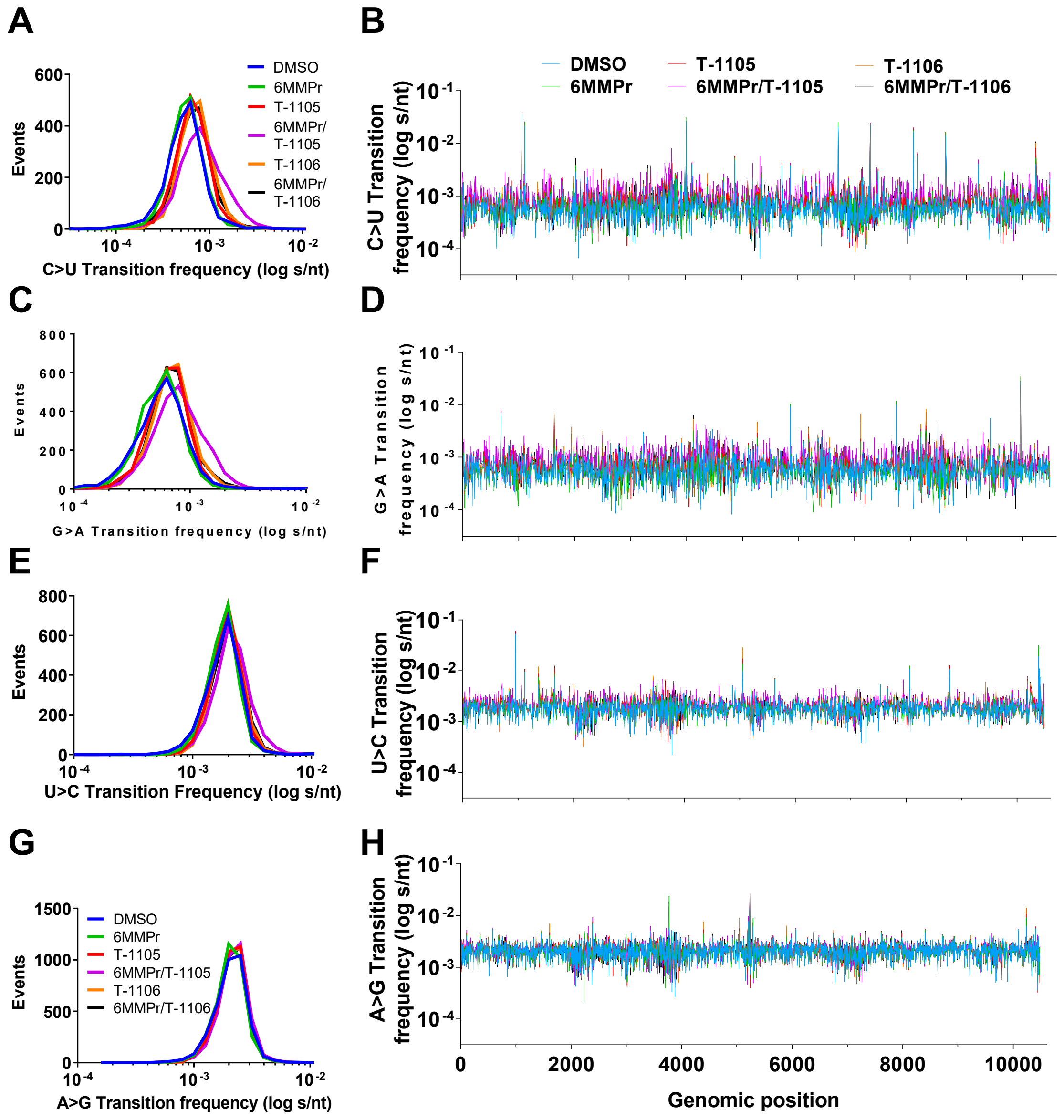

Figure S2. 6MMPr increases the C-U and G-A transition frequencies observed in DENV infected cells treated with T-1105 but not T-1106. Huh7 cells inoculated with DENV (MOI 0.05) were treated with DMSO, 0.1 µM 6MMPr, 52.5 µM T-1105, T-1105/6MMPr combination, 150 µM T-1106 and T-1106/6MMPr combination for 72 hpi. Viral particles from supernatants were concentrated, viral RNA prepared and the number of substitutions analyzed by NGS. In **(A), (C), (E)** and **(G)** the histograms depict the overall transition frequency distribution expressed as the logarithm of the substitution rate (C-U, G-A, U-C and A-G) per nucleotide (log s/nt). Graphs in **(B), (D), (F)** and **(H)** show the C-U, G-A, U-C and A-G transition frequencies expressed as log s/nt across the viral genome. The mean of two independent experiments was used to build the histograms and the plots.

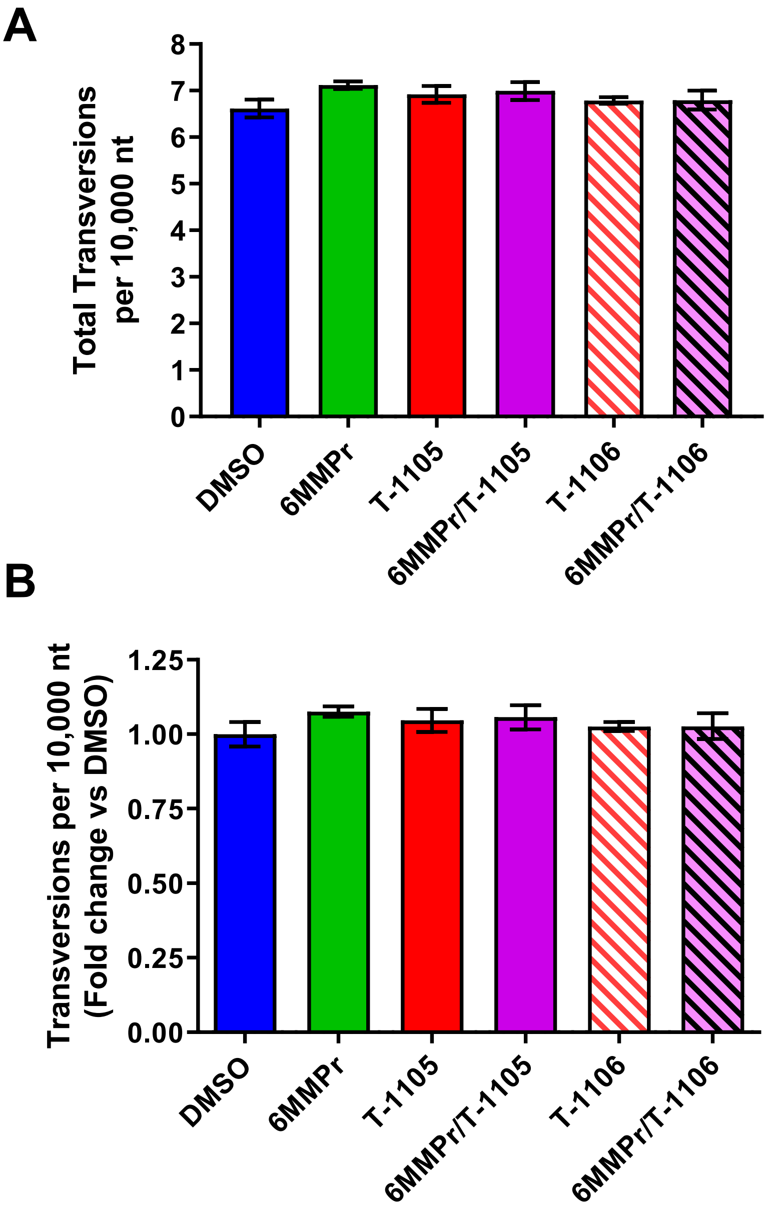

Figure S3. Treatments and combinations do not alter the total number of transversions. Using the data obtained for the NGS analysis, number of transversions were quantified. **(A)** The graph represents transversions mismatches per 10,000 nucleotides (nt). **(B)** Represents the data normalization vs DMSO expressed as fold change.

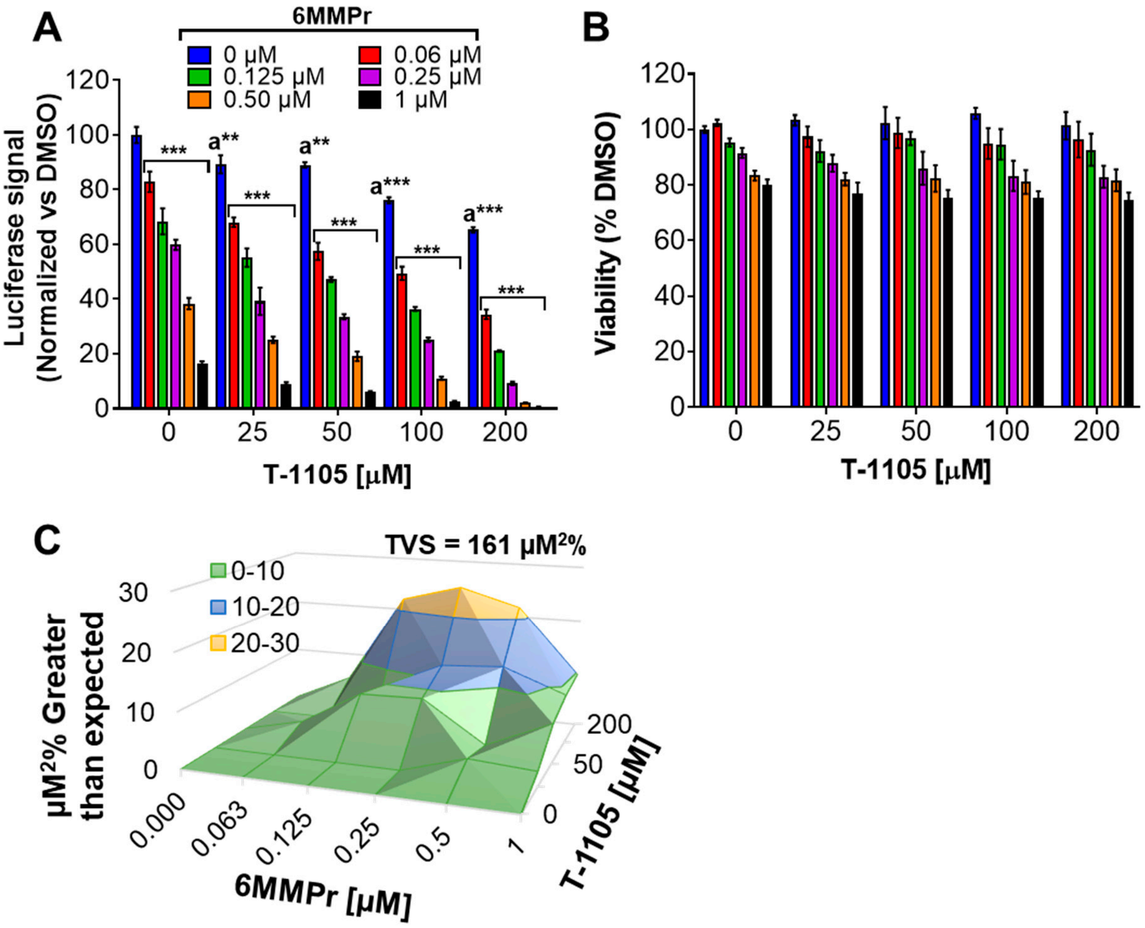

Figure S4. 6MMPr enhances anti-ZIKV effect of T-1105. (A) The bar graph depicts the reduction of luminescence by T-1105/6MMPr co-treatment in HFFs inoculated with ZIKV PAN1 Nluc. Normalized luminescence mean ± SEM from three independent experiments plotted. **(B)** Cell viability analyzed by MTS assay. The graph represents the Mean ± SEM normalized vs DMSO. **(C)** 3-D plots indicating synergistic antiviral effect. Total volume of synergy = TVS. All TVS calculated at 99.9% confidence interval. “a” indicates significant antiviral activity induced by T-1105 alone. * $p<0.05$; ** $p<0.005$; *** $p<0.001$.

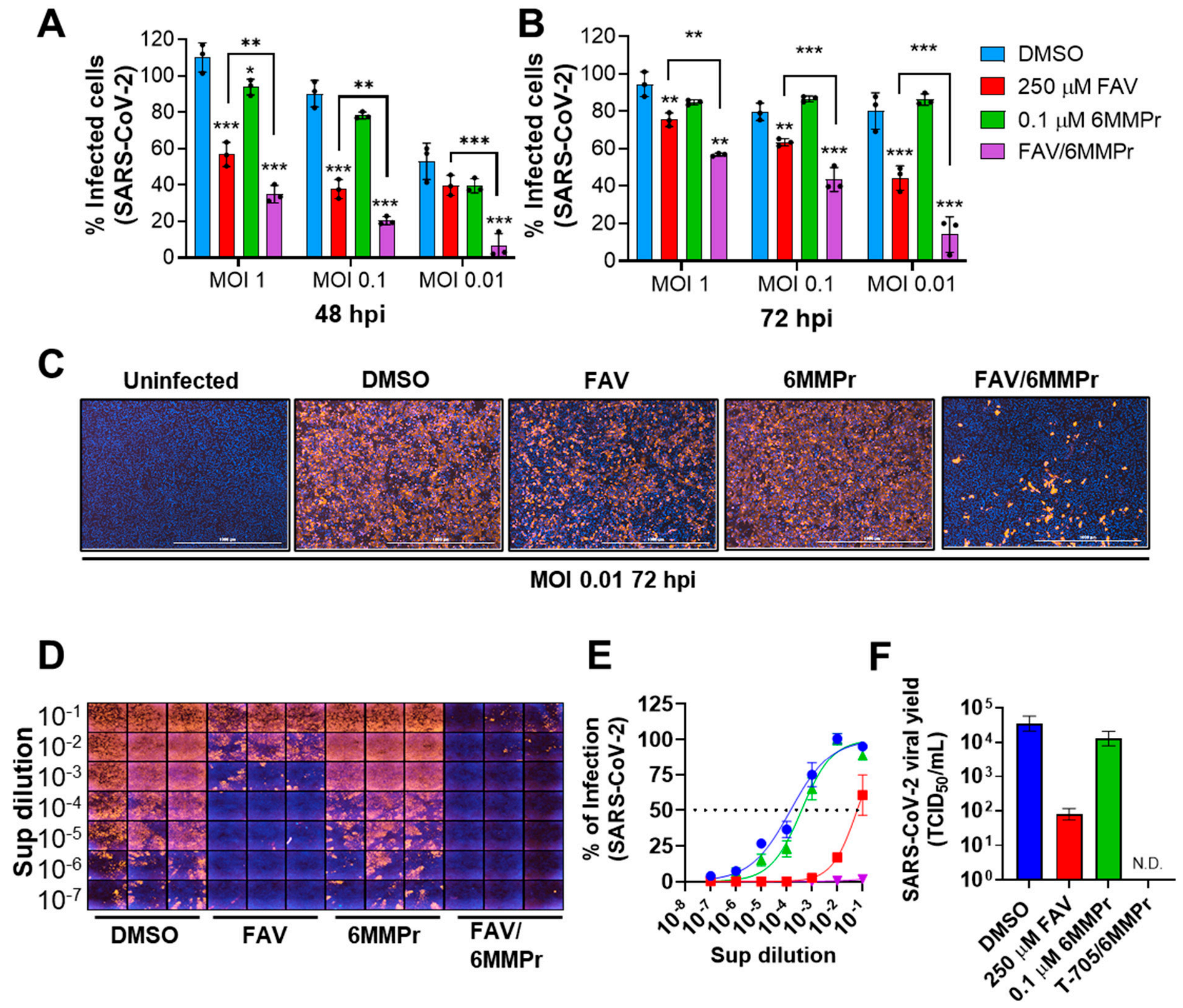

Figure S5. 6MMPr enhances anti-SARS-CoV-2 activity of favipiravir. Vero-E6 cells were inoculated with SARS-CoV-2 at MOI of 1, 0.1 and 0.01 in presence of favipiravir (FAV), 6MMPr, the combination (FAV/6MMPr) or DMSO for 48 **(A)** or 72 hpi **(B)**. The graphs depict the Mean percent of infected cells ±SD of three biological replicates. *, P<0.05; **, P<0.005; ***, P<0.001. Percent infected cells was obtained by immunostaining of viral nucleoprotein (red) and cell nuclei (blue), the representative images correspond to MOI 0.01 72 hpi **(C)**. Supernatants from cells inoculated at MOI of 0.01 for 72 hpi were analyzed for viral yield. 10-fold serial dilutions were used to inoculate Vero-E6 cells. Cells were processed for immunofluorescence **(D)**. Percent infection was calculated for every dilution and used to build viral dose-response curves (**E**) and calculate viral yield expressed as $TCID_{50}$/mL **(F)**.

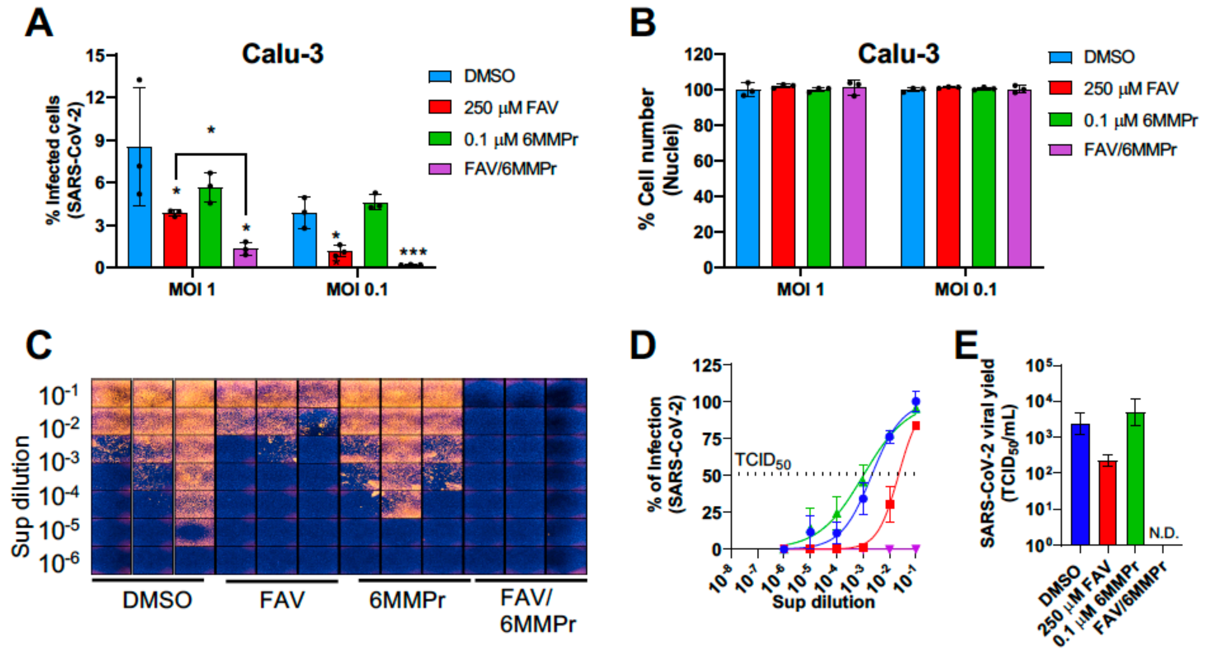

Figure S6. 6MMPr enhances anti-SARS-CoV-2 activity of favipiravir in Calu-3 human lung cells. Calu-3 (**A-E**) cells were inoculated with SARS-CoV-2 at MOIs of 1 or 0.1 in presence of listed compounds. Seventy-two hpi, cells were immunostained for viral nucleoprotein (red) and cell nuclei (blue) and percent infected cells calculated (**A**). Viability (total cell number) was quantified by DAPI staining **(B)**. Supernatants from Calu3 in **A** (MOI 0.1) were analyzed for viral yield **(C-E). (C)** Supernatant inoculated cells processed for immunofluorescence and infected/fluorescent cells depicted in images. **(D)** Percent infection was calculated for every dilution and used to build viral dose-response curves and also expressed as $TCID_{50}$/mL **(E)**. The graphs depict the mean ± SD of samples in triplicate. *, $P<0.05$; **, $P<0.005$; ***, $P<0.00$. N.D. = None detected.

Supplemental Materials and Methods

Cell Lines and Viruses

Baby hamster kidney cells carrying a DENV subgenomic replicon and expressing luciferase (BHK pD2-hRucPac-2ATG30) were obtained from M. Diamond, (Washington University School of Medicine) [1]. Calu-3 cells were a gift from L. Mansky (University of Minnesota). HFFs were obtained from ATCC (CRL-81, CRL-1586 and SCRC-1041, respectively). HFFS, BHK DENV replicon cells were maintained in Dulbecco's modified Eagle's medium (DMEM) supplemented with 10% fetal bovine serum (FBS), 100 IU streptomycin/penicillin per mL and 10 μg/mL plasmocin (InvivoGen). BHK DENV replicon cells were supplemented with 3 μg/mL puromycin (Life Technologies). ZIKV H/PAN/2015/CDC-259359 Nanoluc® reporter virus (ZIKV PAN1 Nluc) was generated as described [2].

DENV replicon assay

BHK cells expressing a DENV replicon were plated in white opaque 96-well plates in the absence of antibiotic selection at $1.5x10^3$ per well. The next day, cells were treated with compounds or DMSO (0.5%). After 72 hours, medium was replaced with a 1:1000 dilution of ViVi-Ren Live Cell Substrate (Promega) in phenol red free DMEM supplemented with 10% FBS. Luminescence was measured with a Molecular Devices M5e plate reader. Mean values of four biological replicates were determined and normalized to the mean value for the wells treated with DMSO alone (0 μM).

ZIKV reporter assay

For ZIKV PAN1 Nluc reporter assays, $1.5x10^4$ HHF cells per well were plated in a 96-well plate. Next day, confluent monolayer was inoculated with ZIKV PAN1 Nluc at a MOI of 0.2 in infection medium for two hours prior to addition of compounds. At 72 hpt, the cells were lysed with 50 μL of 1X luciferase lysis buffer (Promega), incubated 10 min at room temp and stored at -80C. Thirty-five μL of cell lysate was transferred to a white 96-well plate and combined with 35 μL of Nano-Glo® Luciferase Assay substrate diluted 1:50 in Nano-Glo® Luciferase assay buffer (Promega). The reaction was incubated for 2 minutes and luminescence readings were acquired in the Neo 2 plate reader.

SARS-CoV-2 immunofluorescence assay

$1.2x10^4$ Vero-E6 cells or $2.5x10^4$ Calu-3 cells per well were plated in a 96 well plate. Next day the medium was replaced with 50 μL of SARS-CoV-2 infection medium (MEM supplemented with 5% FBS, 100 IU streptomycin/penicillin per mL, 10 mM HEPES, 1X NEA, 1X Glutamax and 1X sodium pyruvate). Cells were treated with compound in 50 μL using infection medium, immediately transferred to the BSL-3 facility and inoculated with 50 μL infection medium containing SARS-CoV-2 at the indicated MOI. 48 or 72 hpi, the cells were fixed with 4% PFA for 30 min and processed for immunofluorescence in a BSL-2 laboratory. Fixed cells were washed three times with PBS and incubated in IF buffer (1X PBS, 0.1% Tween 20) plus 0.3 M glycine and 1% FBS for 30 min. Cells were washed three times with IF buffer and incubated with rabbit anti-SARS-CoV-2 nucleoprotein antibody (Sino Biologicals) in IF buffer + 1% FBS overnight, 4°C. The cells were washed with IF buffer and incubated with goat anti-rabbit Alexa Fluor 555 antibody (Invitrogen) for 2 hours at room temperature. Nuclei were counterstained with DAPI to count the total amount of cells after treatment and as a measure of cell viability. Images were acquired in the Cytation One imaging reader. Total number of DAPI-stained cells and infected cells for each well were quantified in Gen5 software. Percentage of infected cells was determined by dividing the number of infected cells by the total

number of cells per well. Infectious supernatants were analyzed for viral yield by $TCID_{50}$ assays. Briefly, $1.5x10^4$ Vero-E6 cells per well were plated in a 96 well plate. Next day, 10-fold serial dilutions were performed from infectious supernatants in SARS-CoV-2 infection medium (10^{-1} to 10^{-7} dilutions) and used to inoculate Vero-E6 cells in 96-well plates (200 µl). The cells were fixed with 4% PFA at 24 hpi (Vero supernatants) or 48 hpi (Calu-3 supernatants) and processed for IF as above. The percentage of infected cells for each dilution was calculated and plotted into GraphPad prism to perform a non-linear regression analysis, generate infectious dose-response curves and to calculate the $TCID_{50}$/mL for each infectious supernatant.

Data analysis

For Figure S3, parametric or nonparametric tests and the appropriate post-hoc test were applied. If data did not meet assumptions of normality (Shapiro-Wilk test) and equal variance test, then multiple Mann-Whitney U tests were performed. For Figure S7A, one-way ANOVA and Holm-Sidak multiple comparisons were applied. For Figures S1A, S4, S5, and S6 two-way ANOVA with Holm-Sidak's multiple comparison was applied.

References

1. Whitby K, Pierson TC, Geiss B, et al. Castanospermine, a Potent Inhibitor of Dengue Virus Infection In Vitro and In Vivo. Journal of Virology. 2005;79(14):8698-8706.
2. Soto-Acosta R, Jung E, Qiu L, et al. 4,7-Disubstituted 7H-Pyrrolo[2,3-d]pyrimidines and Their Analogs as Antiviral Agents against Zika Virus. Molecules. 2021;26(13):3779.
